# Absence of diabetic retinopathy in a patient who has had diabetes mellitus for 69 years, and inadequate glycemic control: case presentation

**DOI:** 10.1186/1758-5996-1-13

**Published:** 2009-10-05

**Authors:** Jorge Esteves, Carolina Maurente da Rosa, Caroline Kaercher Kramer, Luiz Eduardo Osowski, Stéfano Milano, Luís Henrique Canani

**Affiliations:** 1 Ophtalmology and otorhinolaryngology Division, Hospital de Clinicas de Porto Alegre, Porto Alegre, Brazil; 2 Endocrinology Division, Hospital de Clinicas de Porto Alegre, Porto Alegre, Brazil

## Abstract

The main risk factors for the development and progression of diabetic retinopathy (DR) are chronic hyperglycemia, disease duration and systemic blood pressure. So far chronic hyperglycemia is the strongest evidence concerning the risk of developing DR. However there are some patients with poor metabolic control who never develop this diabetic complication. We present a case of a 73-year-old woman with type 1 diabetes mellitus, diagnosed 69 years ago. The patient is 73 years old, with no evidence of DR, despite poor glycemic control and several risk factors for DR. This case suggests the presence of a possible protection factor, which could be genetic.

## Introduction

Diabetic retinopathy (DR) is the main cause of blindness in individuals aged 20-64 years, and accounts for 7.5% of the causes of adult work disability in Brazil. The risk of blindness is approximately 30-fold higher in people with diabetes mellitus (DM), compared to the population in general [1,2].

The most important risk factors for the development and progression of DR are chronic hyperglycemia, duration of the DM and systemic blood pressure levels [3-9]. Other possible risk factors are dyslipidemia, pregnancy, puberty and local ocular factors such as prior cataract surgery (facectomy) [10].

So far the most solid evidence for the risk of developing DR is chronic hyperglycemia [10-13]. After 20 years duration, there is some degree of DR in almost all patients with type 1 DM, 20% of them severe [14]. In insulin users with type 2 DM, the presence of DR occurs in approximately 84.5% of patients after 15 years of the disease [15].

However, there is great variability in the incidence of DR among patients with DM, and therefore it must be asked why some patients with poor metabolic control remain free of this complication.

We present the case of a 73-year old woman who has had type 1 DM for 69 years, without any evidence of DR, despite inadequate glycemic control and several other risk factors for DR.

## Case Report

**Figure 1:**
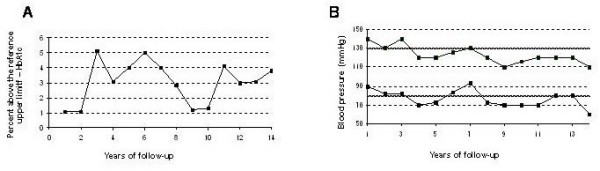
**Patient's long-term glycemic and blood pressure control**. **A**. Evolution of the HbA1c test during the years of follow up (in percentage points above the higher limit of the reference). **B**. Evolution of the control of blood pressure (systolic and diastolic) during the years of follow up compared to the values advocated.

White woman, 73 yrs old, nulliparous, type 1 DM diagnosed at age of 4 yrs, hypertension and dyslipidemia since the age of 44, and diagnosis of hypothyroidism since the age of 40. She smoked 20 cigarettes/day for 37 years. She has used insulin since diagnosis, currently associated with captopril 100 mg/day, simvastatin 40 mg/day, acetylsalicylic acid 200 mg/day and levotiroxine 75 μg/day, and has been regularly followed at the Endocrine Service and Ophthalmology Service at Hospital de Clínicas de Porto Alegre for the last 20 years. Since then, she has maintained inadequate glycemic control with recent mean HbA1c of 9.5% (reference values: 4 - 6.0%). During the follow up period the HbA1c measurement methods were changed at the hospital, and therefore figure 1A presents the values of HbA1c in percentage points above the upper limit of normality of the technique used, showing that the patient remained constantly above the target value. At physical examination during this period, she has had a mean blood pressure, of 124/84 mm Hg (Figure 1B) and maintains a body mass index of 24.2 kg/m^2^. She informed that 23 years ago she had a myocardial infarction (confirmed by a fixed hypoperfusion area in the anterior wall of the myocardium at scintigraphy with dipyramidol), and 4 years ago she was submitted to peripheral artery revascularization surgery (aortofemoral bypass) and amputation of the second toe of the left foot. The latest evaluation of excretion of urinary albumin was 56.6 mg/24 hours (reference value <30 mg/24 hours).

**Figure 2:**
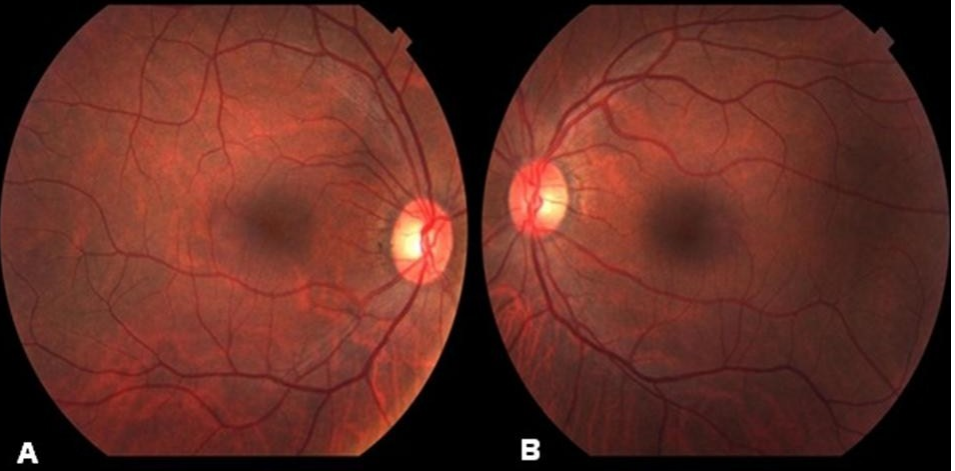
**Eye fundus photograph showing the absence of diabetic retinopathy**. **A **- Right eye **B **- Left eye.

**Figure 3:**
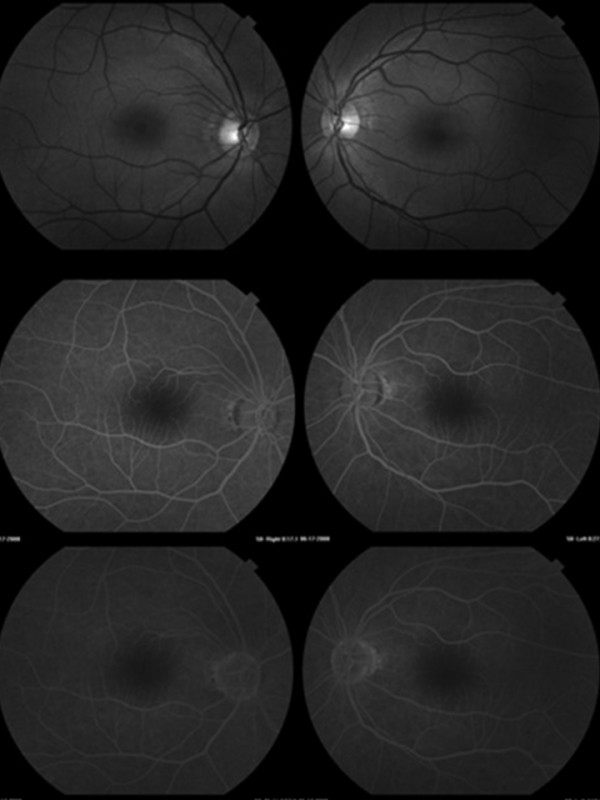
**Study of normal angiofluoresceinography**.

At ophthalmologic examination, a visual acuity of 20/20 J1 was found in both eyes with the best correction. At biomicroscopy of the anterior segment pseudofascia were found in both eyes, with an ophthalmological history of surgery of the cataract in the right eye on June 19, 2002 (extracapsular facectomy) and in the left eye on January 24, 2006 (facoemulsification with intraocular lens implant). Measurement of intraocular pressure (IOP) was performed, always within the limits of normality, with a mean IOP of 14 mmHG in both eyes. At fundus examination of the eye, there was absence of signs of DR (Figure 2). The angiographic examination was also normal (Figure 3).

## Discussion

The case shows the absence of DR in a patient with long duration DM, inadequate glycemic control, hypertension with irregular control and significant macrovascular disease.

Among the risk factors for the development of DM, chronic hyperglycemia and hypertension are the factors with the most solid evidence [1,2,16,17]. In the Wisconsin Epidemiologic Study of Diabetic Retinopathy (WESDR), the initial values of HbA1c were the most important risk factor for the onset and progression of DR [18,19]. Furthermore, hypertension is an important risk factor for the onset and progression of the disease, and in most studies it is an independent risk factor for DR.

The case reported illustrates that, despite the long duration of the disease, a few patients may not develop DR. Besides the duration of DM, age at diagnosis of type 1 DM appears to be related to the presence and severity of DR. Harvey et al observed a greater need for laser therapy in patients with a diagnosis of DM prior to the age of 15 years, and data from the EURODIAB suggest that the pre-pubertal duration of DM is related to the onset of microvascular complications[20,21]. The patient reported with the type 1 DM, besides long duration of the disease presented an early diagnosis, which would further increase the risk of retinal injury.

The role of chronic hyperglycemia in the development of DR has been well established. Maintaining high levels in the HbA1c test is closely related to the development of DR. The accumulated incidence of DR 10 years after type 1 DM has been diagnosed is approximately 90% in patients with HbA1c between 10.2% and 11.5% [22]. Data from WESRD in type 1 DM demonstrate that for every 1% increment in the HbA1c test values, the risk of DR progression increases 1.21 fold [18,19]. The Diabetes Control and Complications Trial (DCCT) and United Kingdom Prospective Study (UKPDS) are randomized clinical trials that demonstrate the efficacy of glycemic control to prevent DR in patients with type 1 and type 2 DM, respectively [3,23]. Every 1% drop in the HbA1c test leads to an estimated reduction of 30% in the risk of microvascular complications [3,23].

The pressure levels also play an essential role in the pathogenesis of DR, increasing intraluminal pressure and worsening the blood-retinal barrier rupture [5-9]. In the WESRD study, diastolic pressure was associated with the presence of any degree of DR in patients with type 1 DM, and it is an independent risk factor for progression to proliferative DR [18,19]. The UKPDS demonstrated that blood pressure control is associated with the reduction of the DR incidence, and the relative risk of DR is 1.5 for systolic BP between 125-139 and 2.8 for systolic BP >140 mm Hg [23].

The presence of DR is associated with cardiovascular mortality both in patients with type 1 DM and in those with type 2 DM. The more severe DR forms being associated with a worse cardiovascular outcome. This evidence suggests that microvascular disease precedes macrovascular disease in a spectrum of a same disease triggered by chronic hyperglycemia, systemic hypertension, dyslipidemia and obesity [11,24].

Even with unequivocal evidence of the role of the risk factors cited previously, in the development of DR, a few as yet unidentified factors appear to counter this, protecting a few individuals from this complication. In the DCCT, approximately 40% of the patients with type 1 DM, without DR at the beginning of the study and HbA1c >9.8% did not present evidence of DR in 10 years of follow up [3].

The clinical course of DR is an example of the effect of the environmental component, in this case hyperglycemia, in the onset of the disease. On the other hand, proliferative DR, the most serious form of DR, affects individuals progressively, reaching a plateau after 20 years of DM. Moreover, intensive control of risk factors does not prevent the development of DR in all the patients [24]. In addition, a few ethnic groups have a higher frequency of DR. Hispanic and African-American patients present greater evidence of DR compared to Caucasian patients. We observed a higher proportion of sever DR among black Brazilian subjects compared to whites [25]. These observations suggest that genetically susceptible patients will develop DR more frequently. Studies of twin siblings with type 2 DM present a concordance for a 95% presence of DR. In patients with type 1 DM, the concordance is lower (67.7%). In both situations, this concordance is much higher than that expected from chance, suggesting a genetic component, and that this is more important in patients with type 2 DM. Studies of non-twin siblings showed that the presence of proliferative DR in one brother with type 2 DM increases the risk for the presence of proliferative DR in his brother with DM around three-fold. In the currently very fashionable studies on candidate genes, the associations of given genetic variants called genetic polymorphisms, with the presence of DR are evaluated. For instance, the genes of aldose reductase, RAGE, VEGF, ACE, *NOS*, *ICAM-1 *and *PPAR*-δ have been considered candidates for DR. A great diversity of results is observed among these genetic studies, with positive and negative associations with DR [2,26-31].

The case reported presents a woman who had had unsatisfactory glycemic control for a long time, hypertension with recent regular control, and severe macroangiopathy manifested by peripheral vasculopathy and cardiovascular disease, without any retinal involvement. This suggests that so far not all factors involved in the pathogenesis of the retinal injury in DM have been identified. More important, they call attention to the existence of locally protective, possibly genetic factor. The identification of this or these factors will enable a better understanding of the pathogenesis of DR and the institution of specific measures for the prevention and treatment of this complication of DM.

## Competing interests

The authors declare that they have no competing interests.

## Authors' contributions section

JE, CMR, LEO, and SM performed ophthalmologic evaluation, charts revision, data analysis and also wrote manuscript. CK and LHC performed clinical evaluation, data analysis, and wrote manuscript. JE and LHC did all manuscript coordination. All authors read and approved the final manuscript

## Consent section

Written informed consent was obtained from the patient for publication of this case report and accompanying images. A copy of the written consent is available for review by the Editor-in-Chief of this journal.
